# Speech and Oromotor Outcome in Adolescents Born Preterm: Relationship to Motor Tract Integrity

**DOI:** 10.1016/j.jpeds.2011.08.055

**Published:** 2012-03

**Authors:** Gemma B. Northam, Frédérique Liégeois, Wui K. Chong, Kate Baker, Jacques-Donald Tournier, John S. Wyatt, Torsten Baldeweg, Angela Morgan

**Affiliations:** 1 UCL Institute of Child Health, London, United Kingdom; 2 Great Ormond Street Hospital for Children NHS Trust, London, United Kingdom; 3 Brain Research Institute, Florey Neuroscience Institutes, Melbourne, Australia; 4 UCL Institute for Women’s Health, London, United Kingdom; 5 Murdoch Childrens Research Institute, Melbourne, Australia; 6 Royal Children’s Hospital, Melbourne, Australia

**Keywords:** CBT, Corticobulbar tract, CST, Corticospinal tract, cUS, Cranial ultrasound scanning, DWI, Diffusion-weighted imaging, FA, Fractional anisotropy, FOC, Focal Oromotor Control, FSIQ, Full-scale IQ, HPI, Hemorrhagic parenchymal infarction, IVH, Intraventricular hemorrhage, MRI, Magnetic resonance imaging, PLIC, Posterior limb of the internal capsule, VMPAC, Verbal Motor Production Assessment for Children

## Abstract

**Objective:**

To assess speech abilities in adolescents born preterm and investigate whether there is an association between specific speech deficits and brain abnormalities.

**Study design:**

Fifty adolescents born prematurely (<33 weeks’ gestation) with a spectrum of brain injuries were recruited (mean age, 16 years). Speech examination included tests of speech-sound processing and production and speech and oromotor control. Conventional magnetic resonance imaging and diffusion-weighted imaging was acquired in all adolescents born preterm and 30 term-born control subjects. Radiological ratings of brain injury were recorded and the integrity of the primary motor projections was measured (corticospinal tract and speech-motor corticobulbar tract [CST/CBT]).

**Results:**

There were no clinical diagnoses of developmental dysarthria, dyspraxia, or a speech-sound disorder, but difficulties in speech and oromotor control were common. A regression analysis revealed that presence of a neurologic impairment, and diffusion-weighted imaging abnormalities in the left CST/CBT were significant independent predictors of poor speech and oromotor outcome. These left-lateralized abnormalities were most evident at the level of the posterior limb of the internal capsule.

**Conclusion:**

Difficulties in speech and oromotor control are common in adolescents born preterm, and adolescents with injury to the CST/CBT pathways in the left-hemisphere may be most at risk.

Children born very prematurely (<33 weeks’ gestation) are at greater risk of speech and language impairments than their full-term peers.^1^ This is a major concern for pediatricians and families, in part because of the known association with academic under-achievement.^2^ Reduced speech quality, poor articulation, and phonological impairments have been detected at school age,^3-5^ and preterm children are more likely to require speech and language therapy. In contrast to language ability, variation in speech performance in this population cannot always be explained by differences in general intelligence.^1,3,6^

Although a number of studies have assessed speech and language abilities, few have clearly distinguished speech intelligibility from expressive and receptive language. This has lead to confusing and often conflicting findings in the literature. Accurate diagnosis of a speech disorder requires assessment in two key areas: speech-sound perception and production and neuromuscular control of the articulators. The latter is of particular relevance to children born preterm because motor symptoms are common in this population,^7^ and speech problems may arise because of a more general motor impairment. Speech intelligibility can also be poor because of a difficulty in producing and perceiving speech sounds. These speech-sound disorders have well-known implications for literacy competence^8^ and have been reported in dyslexia.^9^ To date, few studies have investigated whether speech problems in children born prematurely persist to adolescence, and only one study has examined neuromuscular oromotor abilities in relation to speech outcome in these children.^10^ The current study used a comprehensive battery of expert-rated speech-motor and speech-sound processing tests in a group of adolescents born very prematurely, with a spectrum of brain injury. The significance of any detectable deficits was considered in the context of global motor impairment and general level of cognitive functioning. In addition, the relationship between brain abnormalities and speech deficits was examined with respect to conventional magnetic resonance imaging (MRI) findings and diffusion-weighted neuroimaging (DWI) measures of white matter microstructure, specifically in relation to the primary motor projections (which include the corticospinal and speech-motor corticobulbar tract [CST/CBT]).

## Methods

Fifty adolescents (mean age, 16 ± 1 years) were recruited from a prospective follow-up study of preterm children (<33 weeks’ gestation) at the Neonatal Intensive Care Unit, University College Hospital, London, United Kingdom. In total, 138 children were contacted from birth years 1989 to 1994 (see reference 11 for further details), which included all children with positive cranial ultrasound scanning (cUS) findings at birth (n = 102, excluding 15 with incomplete records). In the final participating sample, 28 children had positive cUS findings (Appendix; available at www.jpeds.com), and 22 children had normal scan results. The mean gestational age was 27.5 ± 2.0 weeks, with a mean birth weight of 1081 ± 385 g. Therefore the proportion of children with brain injury was high (58%, 17 with minor injury and 11 with major injury) compared with the total population born in the same period (29%). Gestational age and birth weight were also significantly lower (by 1 week and by 160 g). However, the group with positive cUS findings was representative of the entire cohort of preterm survivors with brain injury detected at birth for gestation, birth weight, and severity of injury. Thirty term-born adolescents served as control subjects for the DWI measures. These were recruited from siblings and school friends and by local advertisement and were group-matched for age and sex and years of maternal education.

Ethical approval for the study was obtained from Great Ormond Street Hospital for Children/Institute of Child Health Research Ethics Committee, and written informed consent was obtained from all parents or participants, depending on age at assessment.

### General Intelligence

All participants were assessed with the Wechsler Abbreviated Scale of Intelligence.^12^ The mean full scale IQ (FSIQ) score of the preterm group was within 1 SD of the standardized norm mean (FSIQ mean, 91 ± 16; range, 56-120).

### Magnetic Resonance Imaging Abnormalities and Neurologic Status

All children underwent T_1_- and T_2_-weighted MRI scanning at time of follow-up. Scans were reviewed by an experienced pediatric neuroradiologist (W.C.). In the preterm group, 27 children (54%) had “positive” findings (Appendix). Periventricular white matter reduction and reduced size of the corpus callosum were most common (for full details see reference 11). Four adolescents had cerebral palsy (1 bilateral, 3 unilateral), and 13 adolescents had minor neurologic findings (mildly abnormal reflexes or tone, without functional impairment). Neurosensory deficits were found in 3 children (2 with uncorrected visual problems, 1 with visual and hearing impairments).

### Diffusion Weighted Imaging

DWI data were acquired in all participants by using an eddy-current-nulled twice-refocused echo planar imaging sequence^13^ (b-value = 3000s/mm^2^, echo time = 128ms, 60 diffusion-weighted directions, in-plane resolution 2.1 by 2.1 mm^2^, 3-mm slice thickness, 37 contiguous axial slices, acquisition time approximately 9 minutes). Maps of fractional anisotropy (FA) were then computed according to the diffusion tensor model.^14^

### History of Speech/Language and Hearing Impairment

A structured face-to-face interview with parents revealed that 16 children (33%) had received speech and language therapy. This is higher than the median of 5% to 8% reported for either speech or language delay in preschool children.^15^ No children were currently receiving therapy. Ten children required grommets for hearing loss (otitis media with effusion) that resolved spontaneously in all cases by 6 years. One child was excluded because of persistent hearing problems.

### Auditory Processing Screen

The test for Auditory Processing Disorders in Adolescents and Adults^16^ was used to screen for auditory processing deficits. All participating children fell within 2 SDs of the control sample mean.

### Hand Fine-Motor Assessment

To provide a more sensitive neurologic measure, all preterm participants performed the pegboard task from the Zurich Neuromotor Assessment.^17^ This consists of a board containing 12 brass pegs that the child is required to invert and replace in turn. Total time taken was recorded separately for left and right hands.

### Speech Measures

The distinctive production and perception of speech sounds was assessed with the Goldman Fristoe Test of Articulation^18^ and the Comprehensive Test of Phonological Processing, respectively.^19^ The Goldman Fristoe Test of Articulation assesses spontaneous speech production by asking children to name pictures of familiar objects and activities. Recordings were assessed for articulatory or phonological production errors by a speech pathologist (A.M.). The Comprehensive Test of Phonological Processing was used to assess more subtle phonological difficulties by testing the ability to perceive and manipulate speech sounds. Subtests chosen examined familiar words (Phonological Awareness subtest), unfamiliar non-words (Alternate Phonological Awareness subtest), and phonological short-term memory (memory for digits and non-word repetition subtest).

The neuromuscular integrity of the speech system was tested with the Verbal Motor Production Assessment for Children (VMPAC).^20^ The VMPAC assesses neuromuscular control of the articulators during speech and non-speech (“oromotor”) movements. It is reported to have the best overall psychometric properties of any commercially available standardized pediatric motor speech assessment.^21^ The test includes items used to screen for motor-speech disorders (dysarthria and dyspraxia) and is organized into 3 main areas: (1) Global Motor Control; (2) Focal Oromotor Control (FOC); and (3) Sequencing. Two supplemental areas include Connected Speech and Language Control and Speech Characteristics. For each area, a score and classification of impairment (normal, mild, moderate, or severe) can be obtained. The assessment was performed and video-recorded by a trained examiner and later rated by a speech pathologist (A.M.) blinded to neurologic status. This test was used in a subgroup of 36 adolescents (11 individuals with normal cUS findings, 15 with minor injury, and 10 with major injury). Further details of all assessments can be found in Table I (available at www.jpeds.com).

### Neuroimaging Measures

Dilatation of the lateral ventricles at the level of the CST^22^ was measured on T_1_-weighted images in all participants. A ratio was calculated by dividing the width of the ventricle by the width of the hemisphere, both measured from the midline. Intra-rater reliability was good (mean intra-class correlation co-efficient = 0.83 in 21 cases).

The FA of the CST/CBT was used as a measure of its microstructural integrity. FA may reflect fibre density, axonal diameter, or myelination and should be used with caution in regions with a high probability of crossing fibers.^23^ To minimize this, measurement was completed bilaterally at the genu of the internal capsule, at 3 levels in the posterior limb of the internal capsule (PLIC) and in the crus cerebri of the midbrain. Fixed-size regions of interest (3 × 3 voxels; ie, 39.7 mm^3^) were traced on single axial slices of DWI eigenvector maps with in-house software.^24^ Slices were selected according to a standard method.^25^ Mean FA was calculated within each region of interest. There was strong agreement in two raters for mean FA in each hemisphere (intra-class correlation: left = 0.80, right = 0.84 in 25 cases).

### Statistical Analyses

To investigate differences between groups with and without impairment, either the Fisher exact test (for categorical variables) or student *t* test for independent samples (for continuous variables) were used. The mean FA of all 5 regions of the CST/CBT was compared between groups by using a mixed-model ANOVA design with two within-subjects factors: side (left and right hemisphere) and level (genu, PLIC × 3, and midbrain). Spearman rank correlation coefficients (rho) were used to evaluate the relationship between speech and intelligence scores. A stepwise logistic regression analysis was performed to determine the significant predictors (*P* < .05) of a speech impairment.

## Results

Mild articulation errors were detected in only two individuals (4%; both characterized as lateral lisps). Three cases (6%) demonstrated phonological errors or sound substitutions. All remaining errors noted across the group were acceptable productions for children living along the Thames Estuary (“Estuary English”).^26,27^ Errors of this kind were made by 29 children (58%; Appendix).

On measures of phonological awareness, 17 children (34%) scored below average (standardized score <85) on either the word or non-word task. However, in most children, these scores were in line with their FSIQ. Only 4 children (8%) had evidence for a specific phonological awareness deficit (defined as at least 1 SD less than their FSIQ). One child fulfilled the same criterion for a deficit in phonological memory. Both phonological awareness and phonological memory scores were correlated with FSIQ (rho = 0.71 and rho = 0.49, respectively).

None of the children had problems warranting a formal diagnosis of a speech-motor disorder such as dysarthria or dyspraxia. However, more than half of the children assessed (n = 19) did show moderate to severe deficits in one or more areas of the VMPAC. Severe impairments were most common in FOC (Table II). Global Motor Control scores were also reduced, but with further analysis this was revealed to be predominantly caused by failure on oromotor tasks (eg, impaired facial symmetry, tone, and smoothness of movement), which also make up part of the global motor control score. There was a significant association between clinical neurologic findings (minor or abnormal) and deficits on the FOC test (Table III), but neurologic impairment could not account for all cases with FOC deficits. Also, one child with cerebral palsy and 5 children with minor neurologic findings did not show impairments in this area. These findings suggest major difficulties with FOC are common and not fully explained by gross motor abnormalities.

### Magnetic Resonance Imaging Correlates of Speech and Oromotor Deficits

Subsequent investigations of the MRI correlates of speech deficits were therefore restricted to FOC, because this was the most common speech deficit identified and not fully accounted for by the presence of a more global motor impairment.

FOC deficits (including 2 with mild difficulties and 9 rated as severe) were not significantly associated with brain injury detected with cUS at birth, but were associated with an abnormal MRI (positive/equivocal) at follow-up (Table III). There was no significant difference in the asymmetry of lesions detected on MRI (periventricular white matter reduction, high signal on T_2_) or cUS in the groups.

The extent of ventricular dilatation was significantly greater in both hemispheres in children with FOC problems compared with children without FOC problems (Table III). This was not specific to FOC problems; children with abnormal or minor neurologic findings also had greater ratios compared with children with normal examination results (Left: T = 3.03, *P* = .005; Right: T = 2.58, *P* = .02).

The FA of the CST/CBT was reduced in the FOC-impaired group compared with the group with no problems (F[1,34 = 9.1, *P* = .005). In addition, a significant hemisphere-by-group interaction effect was detected (F[1,34] = 6.4, *P* = .02; Table IV). This hemisphere effect was driven by a consistently greater FA reduction in the left versus right PLIC (Figure). These group differences in FA could not be demonstrated when participants were divided on the basis of minor/abnormal neurologic findings or presence of brain injury on conventional MRI.

The FOC-impaired group also took significantly longer than the unimpaired group to complete the pin-turning task with their right hand (36 ± 17 seconds versus 21 ± 5 seconds, respectively; T = 2.75, *P* = .02). No significant group difference was detectable with the left hand (37 ± 19 seconds versus 26 ± 14 seconds, respectively; T = 1.89, *P* = .07). This could not be explained by an over-representation of left-handed participants in the impaired group.

A logistic regression model was computed to determine the significant predictors of FOC deficits with these variables: neurologic outcome (normal versus minor/abnormal), conventional MRI findings (normal versus equivocal/positive), ventricular dilatation ratios (left and right), and mean FA in the left and right CST/CBT. The final model predicted FOC impairment with 81% accuracy (*P* = .001). The only significant independent predictors were neurologic outcome (*P* = .037) and mean FA of the left primary motor tract (*P* = .013).

## Discussion

In this study we carried out a comprehensive evaluation of speech abilities in adolescents born preterm and examined the relationship to conventional MRI findings and DWI investigations of the primary motor pathway. We have shown that although there is no evidence of clinically significant developmental dysarthria, dyspraxia, or a speech-sound disorder in this population, specific difficulties in speech and non-speech oromotor control are common. Furthermore, with DWI, we identified changes along the CST/CBT that were predominantly left-lateralized in the impaired group.

Studies of preterm individuals conducted in early childhood have identified speech deficits, most commonly in speech-sound processing and production.^3-6,28^ Although this study was not prospective, our data suggest that these problems may resolve by adolescence as no significant speech-sound production or phonological awareness deficits could be demonstrated, despite one-third of the sample having received speech and language therapy. In particular, the incidence of lisps and phonological errors was not higher than expected in the general population^29^ and deficits in phonological awareness were entirely consistent with IQ scores.

In contrast, 31% of those assessed showed problems in oromotor and speech-motor control, including difficulties in the precision of individual and combined movements of the lips, jaw, face, and tongue. This is the first study to examine speech and oromotor abilities in adolescence, and our findings are in line with reports in younger children of very low birth weight.^10^ Further, we have shown that although neurologic deficits were significantly more common in this group, the presence of cerebral palsy or minor neurologic findings alone could not predict FOC difficulties (4 of 11 cases had no detectable neurologic deficit).

It is important to consider these findings in the context of the limitations of this study. The sample assessed included a large proportion of individuals who had a positive cUS finding at birth, including 10 with major brain injury. A higher incidence of oromotor problems might therefore be expected. Also, because of the retrospective nature of the study, and limited information about the nature of the initial problems and the amount of speech and language therapy received, the effect of interventions could not be assessed. This should be addressed in future prospective studies.^30^ The possible influence of subtle hearing problems also cannot be excluded, because a detailed audiological examination was not performed. However, there was no evidence for alterations in speech production consistent with a hearing impairment (eg, sibilant distortion), and the impaired group did not perform differently from others on any of the auditory screening tests administered, suggesting this would not have influenced the results.

Despite these limitations, it is encouraging that even in the more severely affected individuals, none were diagnosed with a persistent speech disorder at follow-up. Because this is the most comprehensive study of speech-outcome in this population to date, these findings suggest that speech problems are not a major area for concern in adolescents born prematurely.

CST damage can account for most of the motor deficits in children with cerebral palsy and neuroimaging of the internal capsule plays an important role in predicting motor outcome in such infants.^31^ The link between more subtle motor problems, which are common in preterm children,^7^ and imaging abnormalities is less well established.^32^ In this study, children with speech-motor and oromotor deficits were more likely to show brain injury on MRI at follow-up, but this was not a significant predictor for focal oromotor impairment in the regression analysis. In contrast, DWI measures of diffusion anisotropy in the left primary motor pathway contributed significantly to this prediction (in addition to the presence of neurologic impairment).

The sensitivity of DWI to detect primary periventricular white matter lesions and secondary changes further downstream in the PLIC has been demonstrated previously.^33^ In this sample, such FA reductions may therefore reflect Wallerian degeneration^23^ caused by periventricular white matter injury to the CST/CBT during the neonatal period.^34,35^ This is supported by evidence showing that the main cause for FA reduction in the PLIC in preterm children is caused by an increase in radial diffusivity,^36^ which is in keeping with the diffusion characteristics of secondary degeneration,^23^ and periventricular white matter abnormalities were one of the most common findings on MRI in this group.

DWI of the genu, PLIC, and midbrain therefore may provide a sensitive measure of the degree and laterality of damage to the primary motor pathway not easily visible on cUS or conventional MRI. This conclusion is supported by closer examination of the laterality of major lesions detected on cUS at birth: in the impaired group 3 of 5 children with asymmetrical injury had more severe lesions in the left hemisphere, while the opposite was true (right larger than left) in the 3 asymmetrical cases in the unimpaired group.

These results are not surprising considering the dominant role of the left hemisphere in speech production and articulation^37^ and the recent evidence showing that dysarthria is more common and severe in adults with left rather than right cerebral hemisphere lesions.^38^ However, from a developmental perspective, persistent speech disorders are generally associated with bilateral injury.^39,40^ Our findings are compatible with these data because none of the participants fulfilled the criteria for a clinically significant speech disorder.

In classical descriptions, the CST is said to descend in the PLIC, with representations of the face, hand, and foot arranged from anterior to posterior.^41^ Corticobulbar fibers concerned with speech articulation (representing the face, tongue, and larynx) therefore should lie anteriorly, close to the genu. Therefore we might expect deficits of speech and oromotor control to be most strongly associated with internal capsule abnormalities in this region, but this specificity was not detected. We suspect that this may reflect more generalized damage to the primary motor projections in the left hemisphere, affecting fibers passing through all regions of the posterior limb—including those of the hand and foot. DWI tracking of the individual fiber pathways was not performed here; however, we did show that individuals with oromotor problems were also slower at the hand-motor task, particularly with their right hand (see reference 42). A more detailed examination of neurologic dysfunction may have revealed further evidence for lateralized motor deficits in this group. Although it is conceivable that difficulties in fine hand-motor control might cause real-world difficulties in these children, it is reassuring that residual impairments in FOC do not affect normal speech production in adolescence.

We have shown that everyday speech is generally preserved in adolescents born preterm, even in children with positive MRI findings, neurologic impairment, or a history of speech and language problems. Furthermore, our data suggest that speech abilities may improve with age, in contrast with evidence that intellectual function may decline with time in this population.^43,44^ This could be caused by the timely provision of speech and language therapy or the natural plasticity and reorganization of the developing brain. Our identification of a neural correlate for focal oromotor problems in adolescence raises the possibility of using neonatal imaging to identify individuals at greatest risk of the development of speech problems in early childhood.

## Figures and Tables

**Table I:** Description of speech assessments administered

Area of assessment	Description
1. Oromotor & Speech-motor	VMPAC
a) Global Motor Control	General motor and oromotor control: postural, respiratory and phonatory support, oral reflexes, chewing, swallowing, muscular symmetry of face, strength, tone, range, and smoothness of movement
b) FOC	Deliberate control of the jaw, lips, face, and tongue—both in isolation and in combination
- Non-speech (oromotor) movements	Production of a posture in response to an auditory command Example → “Show me how you bite… and blow”
- Speech movements	Posture control during speech-like utterances, words, phrases, and sentences. Example → “Say… ah um, ah um, ah um, ah um” note: judgement is based on precision of movement not acoustic output
c) Sequencing	Production of speech and oromotor movements described above in the correct sequential order (ie, sequence maintenance)
d) Connected Speech & Language Control	Motor precision in the context of spontaneous language production → formulation of a story based on 4 sequenced pictures
e) Speech Characteristics	Pitch, resonance, vocal quality, loudness, prosody, intonation, and rate
2. Speech-sound	Goldman Fristoe Test of Articulation (a) & Comprehensive Test of Phonological Processing (b & c)
a) Articulation & Phonology	Assesses accuracy in articulation and phonological production of familiar words
b) Phonological Awareness	Segmenting words (Elision) or non-words (Segmenting non-words) into smaller parts → “Say bold without saying /b/” Blending individually presented segments into whole words (Blending Words) or non-words (Blending non-words) → “What word do these sounds make: t-oi?” (correct answer: toy)
c) Phonological Memory	Temporary storage in short term/working memory, and accurate recall of phonological information (Memory for Digits & Non-word Repetition) → “Say it exactly as you hear it….. chasedoolid”

**Figure:**
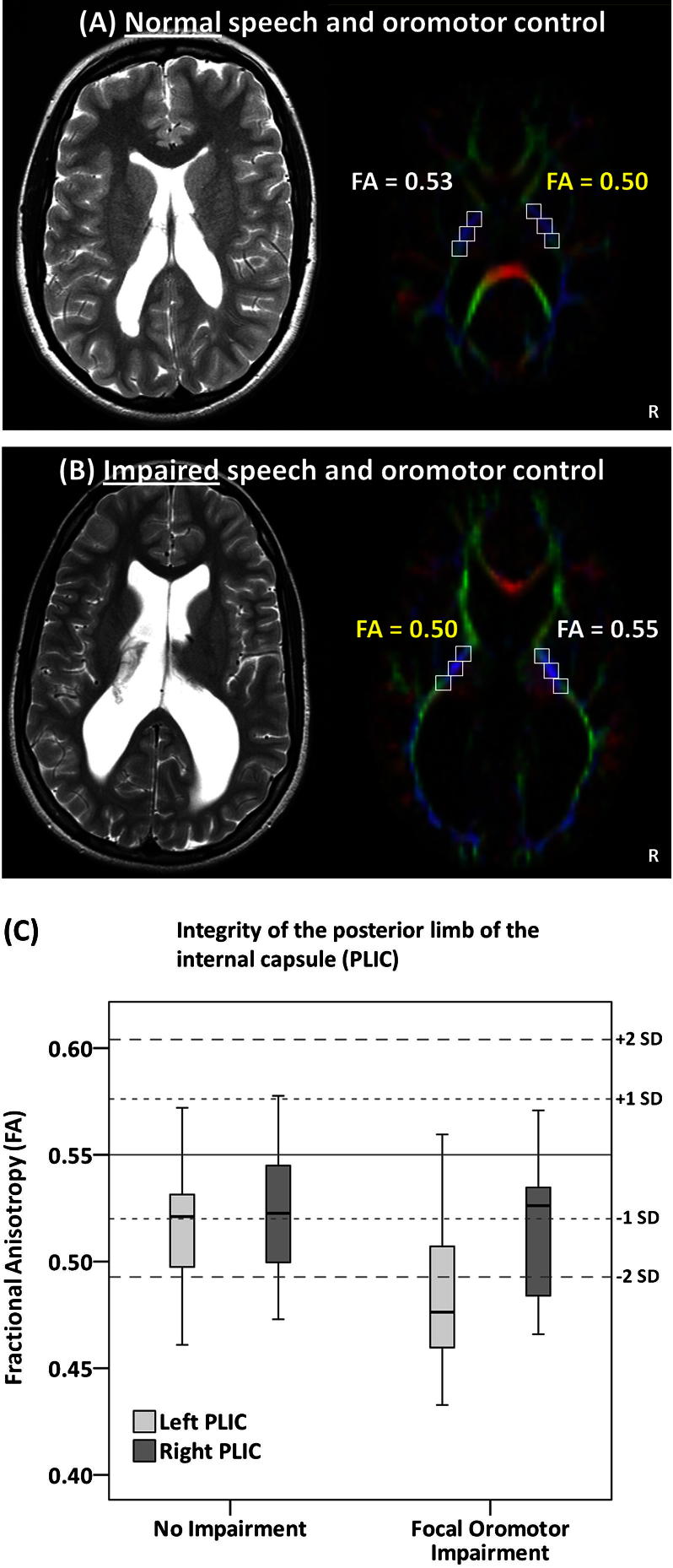
**A** and **B,** Illustrative examples of two preterm children with cerebral palsy. Eigenvector images of case A with normal speech and significantly reduced FA in the PLIC of the right hemisphere and case B with impaired speech and oromotor control and significantly reduced FA in the PLIC of the left hemisphere. **C,** Boxplots of FA values in the PLIC of the left and right hemisphere in preterm adolescents with and without focal oromotor problems. **C,** The *horizontal solid line* represents the mean FA for the term-born control group, with SDs represented by *dotted lines*.

**Table II:** Performance on the VMPAC by subtest

	Normal	Mild/moderate impairment	Severe impairment
Global Motor Control	23 (64%)	6 (17%)	7 (19%)∗
FOC	25 (69%)	2 (6%)	9 (25%)
Sequencing	32 (88%)	3 (8%)	1 (3%)
Connected Speech & Language	31 (86%)	3 (8%)	2 (6%)
Speech Characteristics	25 (69%)	6 (17%)	5 (14%)

∗ Impairment predominantly because of failure on focal oromotor tasks.

**Table III:** Comparison between focal oromotor impaired and unimpaired preterm groups

	Unimpaired focal oromotor (n = 25)	Impaired focal oromotor (n = 11)	Statistical comparison
cUS findings none (minor, major)∗	7, (13, 5)	4, (2, 5)	*P* = .45
Neurologic outcome normal (minor, abnormal)∗	18, (6, 1)	4, (4, 3)	*P* = .05
Previous speech and language therapy	5	6	*P* = .05
Positive MRI	17 (68%)	11 (100%)	*P* = .04
Grey matter abnormality	1 (4%)	5 (45%)	
High-signal on T_2_	7 (28%)	5 (45%)	
Corpus callosum reduction	10 (40%)	10 (91%)	
Periventricular WM reduction	10 (40%)	9 (82%)	
Ventricular dilatation ratio†			
Left	0.27 ± 0.04	0.32 ± 0.07	*P* = .01
Right	0.26 ± 0.04	0.31 ± 0.05	*P* = .01

*WM*, white matter.

∗ Groups in brackets were collapsed and treated as one group for comparison with the normal or none group.

† Values were converted with a logarithmic transformation before statistical comparison. One case was excluded due to massive ventricular dilatation.

**Table IV:** Comparison of microstrcutural measures (FA) of the CST/CBT in the focal oromotor impaired and unimpaired groups using two-sample *t* tests (two-tailed)

CST region of interest	Unimpaired focal oromotor (n = 25)	Impaired focal oromotor (n = 11)	Statistical comparison
1. Genu			
Left	0.463 ± 0.043	0.423 ± 0.073	T = 2.05, *P* = .05
Right	0.477 ± 0.045	0.436 ± 0.060	T = 2.23, *P* = .03
2. PLIC (ant. portion)			
Left	0.491 ± 0.039	0.453 ± 0.055	T = 2.42, *P* = .02
Right	0.489 ± 0.047	0.488 ± 0.059	T = 0.07, *P* = .95
3. PLIC (mid portion)			
Left	0.548 ± 0.036	0.509 ± 0.076	T = 2.06, *P* = .05
Right	0.550 ± 0.040	0.541 ± 0.076	T = 0.44, *P* = .66
4. PLIC (post. portion)			
Left	0.565 ± 0.055	0.518 ± 0.058	T = 2.36, *P* = .02
Right	0.580 ± 0.044	0.552 ± 0.067	T = 1.45, *P* = .16
5. Midbrain			
Left	0.465 ± 0.084	0.378 ± 0.111	T = 2.58, *P* = .01
Right	0.446 ± 0.076	0.376 ± 0.094	T = 2.17, *P* = .05

Mean values and SDs are given in each group. These post-hoc tests did not survive correction for multiple comparisons.

## Appendix

Speech errors: Phonological errors detected: stopping a fricative (ie, substituting the fricative /v/ with a plosive or “stop” sound /b/ in the word initial position). Estuary English errors included: insertion of a glottal stop replacing word-final plosive /t/; gliding /w/ for /r/ in word-initial positions; and fronting voiced fricative /th/ to voiced fricative /v/ and voiceless fricative /th/ to voiceless fricative /f/.

Cranial ultrasound: A positive cUS finding in the first few weeks of life was defined as either: (1) intraventricular hemorrhage grade I (IVH-1), in which bleeding is restricted to the region of the germinal matrix; (2) intraventricular hemorrhage grade II (IVH-2), indicating the presence of blood within the ventricular system; (3) blood within and distending the lateral ventricles (IVH-3); (4) hemorrhagic parenchymal infarction (HPI)—increased echodensities in the periventricular white matter associated with an IVH; or (5) periventricular leukomalacia —cystic lesion within the periventricular white matter, not preceded by HPI at the same site. cUS findings were categorized as minor or major. Minor injury included IVH-1 and IVH-2. Major injury included IVH-3, HPI and periventricular leukomalacia.

Qualitative MRI assessment: All scans were evaluated using a standardized protocol by an experienced pediatric neuroradiologist (W.C.) who was blinded to previous ultrasound scanning findings. Scans categorized as “positive” included findings considered to be consistent with preterm birth. Those categorized as “equivocal” showed isolated, potentially incidental abnormalities, such as single small lesions in the subcortical gray or white matter.
